# Aberrantly Expressed Small Noncoding RNAome in Keloid Skin Tissue

**DOI:** 10.3389/fgene.2022.803083

**Published:** 2022-04-13

**Authors:** Chuang Yin, Chuandong Wang, Chen Wang

**Affiliations:** ^1^ Department of Plastic and Reconstructive Surgery, Shanghai Ninth People’s Hospital, Shanghai Jiao Tong University School of Medicine, Shanghai, China; ^2^ Department of Orthopedic Surgery, Xinhua Hospital Affiliated to Shanghai Jiao Tong University School of Medicine, Shanghai, China

**Keywords:** keloid, miRNA, piRNA, snoRNA, snRNA, rasiRNA

## Abstract

The skin is an organ that protects against injury and infection but can be damaged easily. Wound healing is a subtle balance which, if broken, can lead to keloid formation. Small noncoding (nc) RNAs can be of “housekeeping,” for example, ribosomal RNAs and transfer RNAs, or “regulatory,” for example, microRNAs (miRNAs or miRs), small nucleolar RNAs (snoRNAs), and P-element–induced Wimpy testis (PIWI)-interacting RNA (piRNA) types. We examined five types of small ncRNAs [miR, piRNA, snoRNA, small nuclear (sn) RNA, and repeat-associated small interfering RNA (rasiRNA)] in keloid skin tissue (KST) using sequencing and real-time reverse transcription-quantitative polymerase chain reaction. All comparisons were made in relation to expression in normal skin tissue (obtained by abdominoplasty). The expression of three piRNAs was upregulated, and the expression of six piRNAs was downregulated in KST. The expression of 12 snoRNAs was upregulated, and the expression of two snoRNAs was downregulated in KST. The expression of two snRNAs was downregulated in KST. The expression of 18 miRs was upregulated, and the expression of three miRNAs was downregulated in KST. The expression of one rasiRNA was upregulated, and the expression of one rasiRNA was downregulated in KST. We revealed the differential expression of small ncRNAs in KST, which may aid the development of new treatment for keloids.

## Introduction

The skin comprises the epidermis, hypodermis, and dermis. The latter is separated into a papillary layer and reticular layer, with sweat glands and hair follicles interspersed within them (Schulze et al., 1997). The papillary layer is composed mainly of microvascular vessels, nerve endings, and different cell types (Stücker et al., 2002). An extracellular matrix (ECM) enriched with collagen is the main component of the reticular layer (Marcos-Garcés et al., 2014). Fibroblasts account for the largest proportion of cells in both layers, but they are slightly different. Fibroblasts in the papillary layer are spindle-shaped, highly proliferative, and mostly synthesize proteoglycans (Pageon et al., 2012). Fibroblasts in the reticular layer are stellate-shaped, show low proliferation, and mostly synthesize elastin collagen (Schafer et al., 1985).

Wound healing is a subtle balance which, if broken, can lead to undesirable results such as keloids (Andrews et al., 2016). A keloid is a benign hyperplastic disease with excess ECM deposition (Lee and Jang, 2018). After a keloid has been formed, the tissue structure is completely different from that of the normal skin. The dermis in the normal skin contains a low density of irregularly arranged collagen (Bagabir et al., 2012). In a keloid, the dermis is expanded significantly and can be divided approximately into three layers. The superficial dermis is similar to the granulation in normal scars. In the middle layer, there are many activated fibroblasts and lymphocytes surrounded with type-III collagen in the border and static fibroblasts in the center. The deep dermis is full of degenerated and necrotic dermal cells (Jiao et al., 2017).

Approximately 98% of human DNA does not code protein. Previously thought to be of little interest, this entire DNA was found to be biologically active by the ENCODE Project (Carninci et al., 2005). Part of this non–protein-coding DNA is transcribed consistently in noncoding (nc) RNA. The latter comprises “housekeeping ncRNA,” for example, ribosomal (r) RNAs and transfer (t) RNAs, and “regulatory RNAs,” for example, microRNAs (miRNAs or mIRs), small nucleolar RNAs (snoRNAs), and P-element–induced Wimpy testis (PIWI)-interacting RNAs (piRNAs) (Djebali et al., 2012)]. Research on regulatory RNAs has made considerable progress, and solid evidence of regulatory RNAs has been found in several biological pathways (Xue et al., 2020).

Among these regulatory RNAs, miRs are the most studied. A mature miR molecule, through an RNA-induced silencing complex (RISC), targets a specific messenger (m) RNA sequence to prevent translation or to initiate mRNA degradation (Lu and Rothenberg, 2018). Evidence of miR dysregulation has been found in every step of disease formation from development to drug resistance (Mollaei et al., 2019; Van Meter et al., 2020). Several studies have found that miRs have a regulatory role in keloid formation. miR-92a (Yu et al., 2018) and miR-196a (Kashiyama et al., 2012) can contribute to collagen expression. miR-21 (Wang et al., 2012) is associated with the proliferation and migration of fibroblasts. miR-221 and miR-222 (Stadnik et al., 2021) have been found to be involved in cell activities in response to a mechanical force.

piRNAs have very diverse nucleotide sequences in ncRNAs. piRNAs were first discovered in the germ cell lines of mice (Betel et al., 2007). piRNAs can be divided approximately into three groups based on derivation: transposon, long noncoding (lnc)RNA, or mRNA. Only transposon-derived piRNAs have been studied deeply (Czech et al., 2018). piRNA functions are not clear, but researchers have found differences in the expression between healthy controls and people with different types of cancer (Liu et al., 2019). piRNA-651 has been reported to participate in the pathophysiology of hepatocellular carcinoma through the protein kinase B signaling pathway (Cheng et al., 2011). The upregulated expression of piRNA-823 in hepatic stellate cells accounts for liver fibrogenesis (Tang et al., 2018) and the differential expression of piRNAs in cardiac fibroblasts (Vella et al., 2016), which suggests the potential role of piRNAs in the fibrogenesis observed in keloids. Also, X. Zhou and colleagues observed piRNAs to be closely involved with wound healing (Chen et al., 2020).

The function of small nucleolar (sno) RNAs in post-transcriptional modification of rRNAs within the nucleolus is well documented (Xing and Chen, 2018). However, in recent years, studies about snoRNAs in other types of cellular regulation have emerged, such as miR-like gene silencing and miR “sponging” beyond the nucleolus (van der Werf et al., 2021). Several investigators have found a clear relationship between snoRNAs and cancer (Askarian-Amiri et al., 2011; Li et al., 2015). Numerous lncRNAs have been shown to regulate miRs in keloid formation through RNA sponging (Duan et al., 2020), so we speculated that snoRNAs might be regulators.

A small nuclear RNA (snRNA) is a small RNA of length of ∼150 nucleotides. Its main function is processing heterogeneous nuclear RNA. snRNAs with diverse expression across tissue and cancer samples have been reported, which can induce differences in alternative splicing (Dvinge et al., 2019).

In epigenetics studies of keloids, researchers have focused on DNA methylation and ncRNA regulation (Stevenson et al., 2021). In recent years, the abnormal expression of miRs and the interaction between lncRNAs and miRs in keloids have been studied widely. There is ample evidence that miRs regulate cell behaviors such as apoptosis and ECM production in keloid formation (Liu et al., 2014; Liu et al., 2016; Huang et al., 2018). Here, we examined the four types of ncRNAs (miR, piRNA, snoRNA, and snRNA) in keloids. We hoped to reveal the differential expression, which may aid the development of new treatment for keloids.

## Materials and Methods

### Ethical Approval of the Study Protocol

The study protocol was approved by the ethics committee of Shanghai Ninth People’s Hospital (Shanghai, China). Written informed consent was obtained from each patient recruited. Tissue collection was in compliance with the “Code for Proper Secondary Use of Human Tissue” in accordance with the Declaration of Helsinki 1964 and its later amendments.

### Patients and Samples

Keloid skin tissue (KST) and normal skin tissue (NST) were obtained from patients treated at Shanghai Ninth People’s Hospital. Keloids were diagnosed by histology (Supplementary Table S1). No patients received any treatment before the surgical procedure. All normal skin was obtained from abdominoplasty. KST and NST were excised carefully. Then, 4-mm punch biopsies were taken from every sample. The sample tissue was flash-frozen using liquid nitrogen, placed immediately in liquid nitrogen, and stored at −80°C.

### Ribonucleic Acid Isolation

Total RNA was isolated from KST and NST using the TRIzol™ reagent (catalog number: 15596018; Life Technologies, Carlsbad, CA, United States) according to manufacturer’s instructions. Briefly, 100 mg of KST or NST, respectively, was taken, and 1 mL of TRIzol reagent was added. The tissue was homogenized with a tissue homogenizer. Then, 200 μL of chloroform was added, and the mixture was shaken and mixed evenly. After centrifugation, the supernatant was removed, and isopropanol was added to precipitate RNA. Then, 75% ethanol solution was added for washing; the precipitate was retained after centrifugation, and diethyl pyrocarbonate water was added to dissolve RNA.

### Real-Time Reverse Transcription-Quantitative Polymerase Chain Reaction

Using 1 μg of total RNA, The First Strand cDNA Synthesis (Poly A Tailing; B532451; Sangon Biotech, Beijing, China) and PrimeScript™ RT Master Mix (Perfect Real Time) (RR036A; Takara Biotechnology, Shiga, Japan) were employed to reverse RNA into complimentary DNA, respectively, according to product instructions. U6 was used as an endogenous control for the normalization of miRNAs and piRNAs qPCR data, and GAPDH was used as the endogenous control for the normalization of snoRNA and snRNA qPCR data. Then, according to the product manual, SYBR™ Premix Ex Taq (RR420A; Takara Biotechnology) was used for PCRs, and the gene expression was calculated using the 2^−△△CT^ method. The primers used in this study are listed in Supplementary Table S2.

### Sequencing of Small Ribonucleic Acids

After the samples passed the test, the small RNA sample prep kit was used to construct the library. After library construction, Qubit 2.0 (Invitrogen, CA, United States) was used for preliminary quantification. The library was diluted to 1 ng/µL. TruSeq PE Cluster Kit v3-cBot-HS (Illumina, San Diego, CA, United States) was employed to generate clusters on cBot (Illumina). Then, the sequencing program (se50) was run on the Illumina sequencing platform to obtain sequencing reads. The sequencing data of small RNAs were run on the Illumina platform. Then, we measured the expression of small RNAs in species by comparing it with the reference genome. Filtered data were compared with the representative sequences in the Rfam database (https://rfam.xfam.org/) to annotate other ncRNA sequences. For the sequencing data of small RNAs, we selected rRNA, long intergenic noncoding (linc) RNA, tRNA, snRNA, snoRNA, and small bacterial (s) RNA in the Rfam database to annotate the reads obtained by sequencing. We judged whether there was a significant difference in the expression between the two groups. For experiments with biological duplication, we used DESeq (R Institute for Statistical Computing, Vienna, Austria) for analyses; for experiments without biological duplication, we used edgeR (https://bioconductor.org/) for analyses. Finally, genes with p-adjusted <0.05 and differential multiple ≥1.5 were selected as differentially expressed small RNAs.

### Cell Culture

Keratinocytes were extracted from the epidermis using 0.25% trypsin (Invitrogen, San Diego, CA, United States) at 37°C for 30 min. All cells were filtered through a cell strainer (70 µm; Merck Millipore, Burlington, MA, United States). Primary keratinocytes were incubated in Keratinocyte SFM (1X) (ScienCell, San Diego, CA, United States) supplemented with 10% fetal bovine serum (Biological Industries Cromwell, CT, United States) and 1X penicillin-streptomycin-fungizone (PSF; Life Technologies) for 48 or 72 h at 37°C to allow the cells to adhere to the culture dishes. Non-adherent cells were washed out with phosphate-buffered saline (PBS), and the remaining cells were subcultured or collected for the following analysis at approximately 80–90% confluence to avoid contact inhibition and differentiation. Cells at up to passage 3 were used for analyses.

### Statistical Analyses

Data are the mean ± SD of three independent experiments. The Student’s *t*-test was used to compare the difference in data from two groups using Excel™ within Office™ 2017 (Microsoft, Redmond, WA, United States). *p* < 0.05 was considered significant.

## Results

### Aberrant Expression of piRNAs Within Keloid Skin Tissue

**FIGURE 1:**
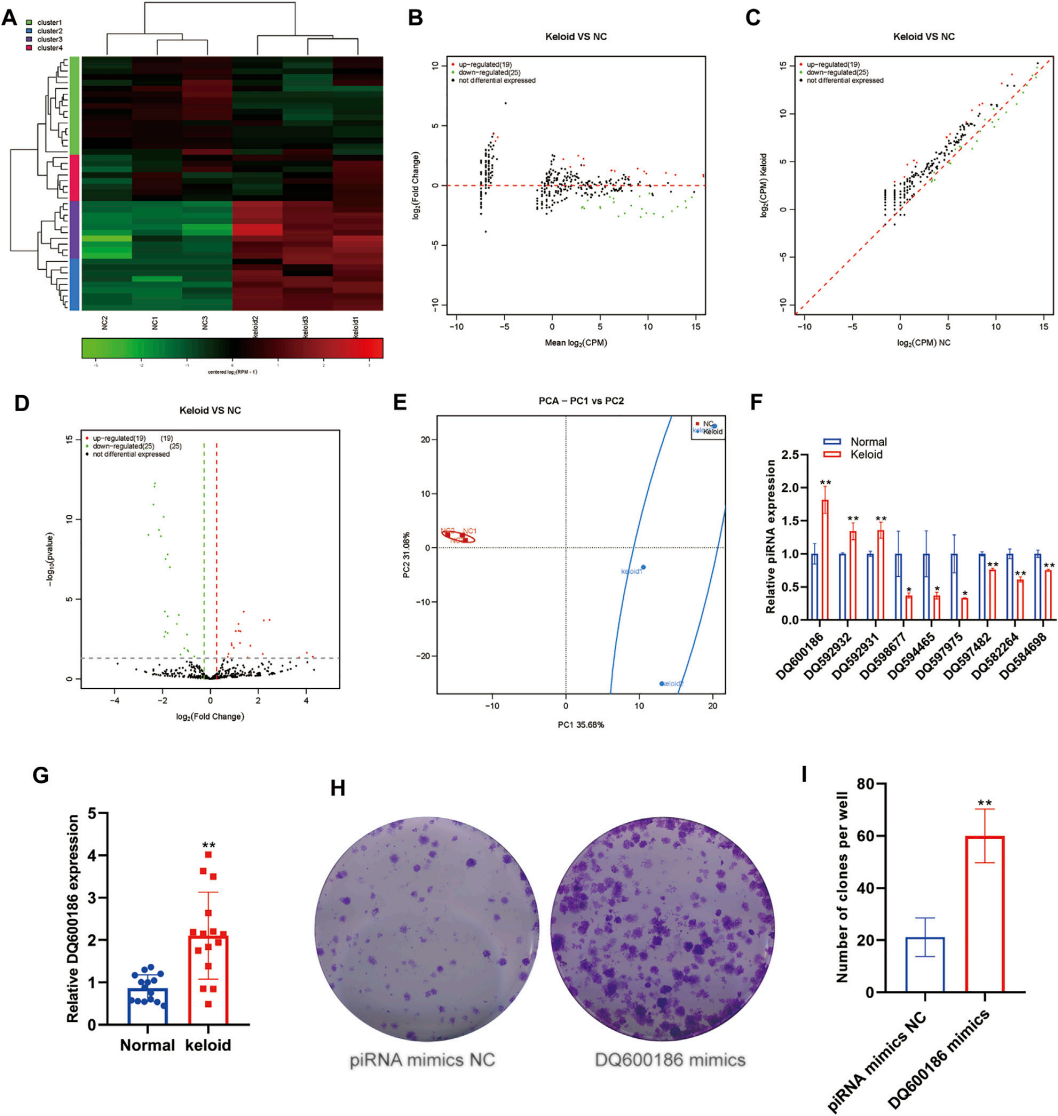
Aberrantly expressed piRNA within the keloid tissue. **(A)** Heatmap showed the expression of piRNA within the keloid tissue and normal tissue. **(B)** MA map showed the expression of piRNA within the keloid tissue and normal tissue. **(C)** Scatter map showed the expression of piRNA within the keloid tissue and normal tissue **(D)** Volcano map showed the expression of piRNA within the keloid tissue and normal tissue **(E)** PCA analysis of the expression of piRNA within the keloid tissue and normal tissue. **(F)** qPCR detection of the expression of piRNA in the keloid tissue and normal tissue. * indicated *p* ＜ 0.05 compared to the normal skin tissue, and ** indicated *p* ＜ 0.01 compared to the normal skin tissue.

piRNA is a type of small ncRNA isolated from mammalian germ cells. This type of small ncRNA can interact with members of the PIWI protein family. However, no study has reported the piRNA expression in keloid tissue. We first measured the changes in the expression of piRNAs in NST and KST. The sequence of small ncRNAs showed different expressions of piRNAs in the KST and NST. Bioinformatics analysis showed that compared with NST, the expression of 19 piRNAs was upregulated significantly, and the expression of 25 piRNAs was downregulated significantly, in KST. piRNAs that are downregulated in keloids have, on average, higher *p* values, predicting that there may be a mechanism for this piRNA to suppress the cellular activity (Figures 1A–D). Principal component analysis (PCA) showed that piRNA expressions in KST and NST had significantly different characteristics. The PCA plot demonstrated that the group of normal skin was highly clustered together. Both PC1 (35.68%) and PC2 (31.08%) could significantly distinguish the two groups, but the lack of concentration on PC2 in the keloid group predicts that there may be multiple directions of variation for this piRNA in keloid and multiple regulatory mechanisms for disease formation (Figure 1E). RT-qPCR revealed that expressions of DQ600186, DQ592932, and DQ592931 were upregulated, and expressions of DQ598677, DQ594465, DQ597975, DQ597482, DQ582264, and DQ584698 were downregulated, in KST compared with that in NST (Figure 1F).

### Aberrant Expression of snoRNAs Within Keloid Skin Tissue

**FIGURE 2:**
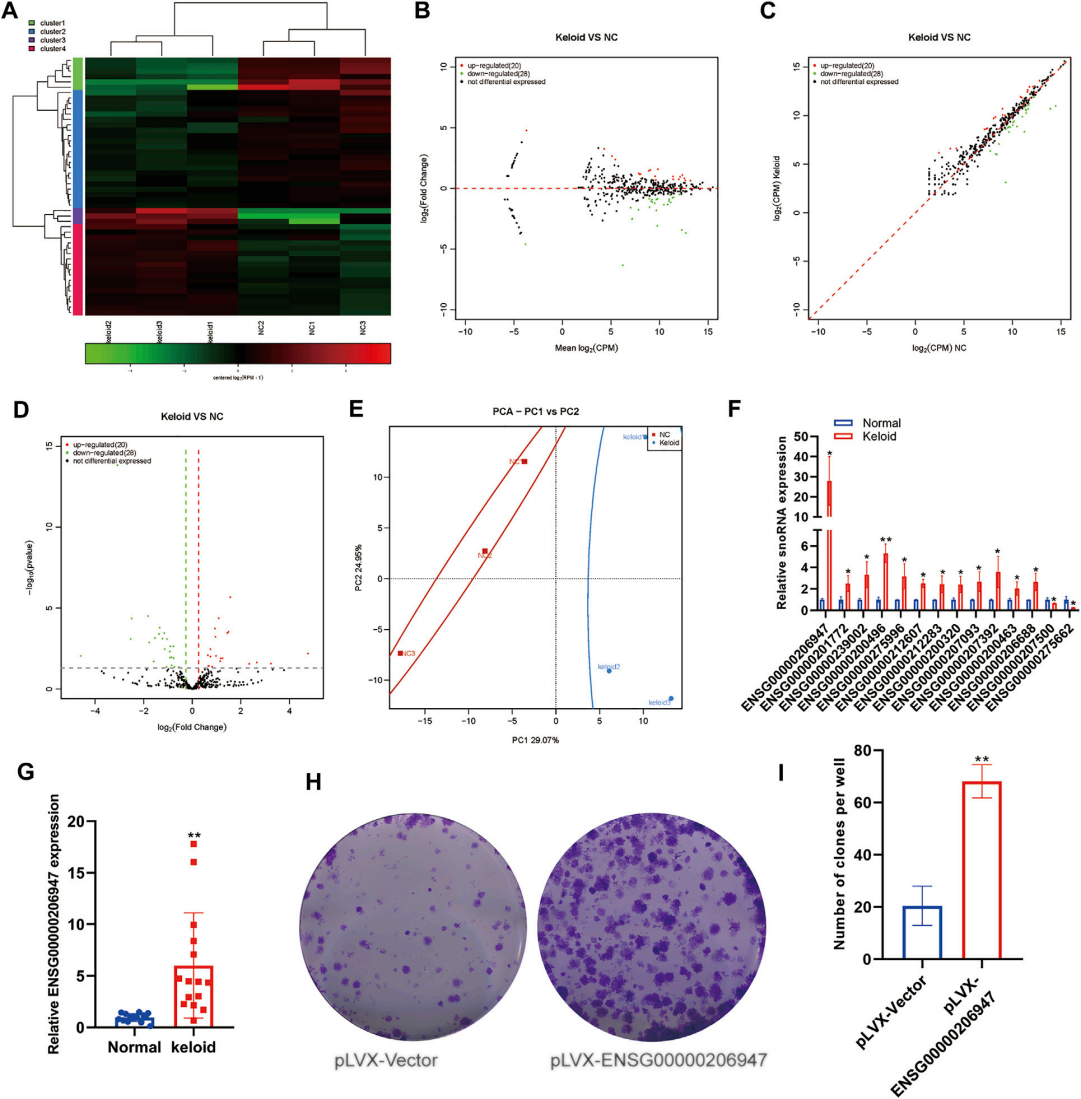
Aberrantly expressed snoRNA within the keloid tissue. **(A)** Heatmap showed the expression of snoRNA within the keloid tissue and normal tissue. **(B)** MA map showed the expression of snoRNA within the keloid tissue and normal tissue. **(C)** Scatter map showed the expression of snoRNA within the keloid tissue and normal tissue. **(D)** Volcano map showed the expression of snoRNA within the keloid tissue and normal tissue. **(E)** PCA analysis of the expression of snoRNA within the keloid tissue and normal tissue. **(F)** qPCR detection of the expression of snoRNA in the keloid tissue and normal tissue. * indicated *p* ＜ 0.05 compared to the normal skin tissue, and ** indicated *p* ＜ 0.01 compared to the normal skin tissue.

snoRNA is also a type of small ncRNA, with a length of approximately 60–30 nucleotides. Initial studies revealed snoRNAs to be located mainly in the nucleolus, that they were related to the processing and modification of rRNA, and that their function was relatively simple. Subsequently, sequencing data showed that snoRNAs, in general, had a high expression in tumors, and some studies showed snoRNAs to be involved in disease processes. However, no study has reported the snoRNA expression in KST. We evaluated changes in the expression of snoRNAs in NST and KST. The sequence of small ncRNAs showed different expressions of snoRNAs in KST and NST. Bioinformatics analysis showed that compared with NST, the expression of 20 snoRNAs was upregulated significantly, and the expression of 28 snoRNAs was downregulated significantly, in KST. Despite the small number of snoRNAs annotated as differentially expressed, there were on average larger fold changes among the downregulated snoRNAs, predicting that these few variants may be more closely involved in the regulation of keloid formation (Figures 2A–D). PCA showed that snoRNA expressions in KST and NST had significantly different characteristics in PC1 (29.07%) but lack of individuality in PC2 (24.95%) (Figure 2E). This result may come from the property of snoRNAs as nuclear RNAs involved in the regulation of most cellular basal functions, such that a small number of variant snoRNAs in keloid could not be identified with sufficient differential expressions. RT-qPCR revealed that the expression of 12 snoRNAs was upregulated, and the expression of two snoRNAs was downregulated, in KST compared with that in NST (Figure 2F).

### Aberrant Expression of snRNAs Within Keloid Skin Tissue

snRNAs are the main components of RNA spliceosomes during post-transcriptional processing of RNA in eukaryotes. However, no study has reported on the snRNA expression in KST.

**FIGURE 3:**
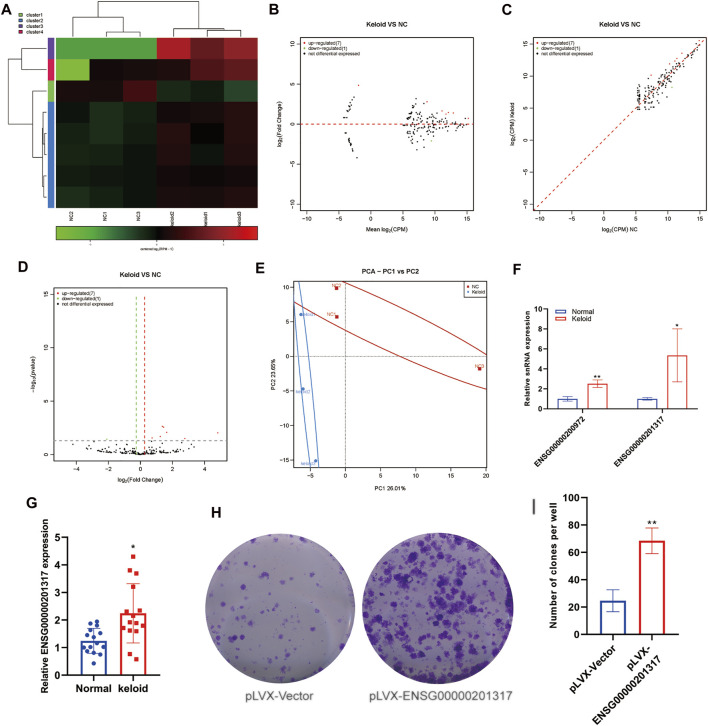
Aberrantly expressed snRNA within the keloid tissue. **(A)** Heatmap showed the expression of snRNA within the keloid tissue and normal tissue. **(B)** MA map showed the expression of snRNA within the keloid tissue and normal tissue. **(C)** Scatter map showed the expression of snRNA within keloid tissue and normal tissue. **(D)** Volcano map showed the expression of snRNA within the keloid tissue and normal tissue. **(E)** PCA analysis of the expression of snRNA within the keloid tissue and normal tissue. **(F)** qPCR detection of the expression of snRNA in the keloid tissue and normal tissue. * indicated p＜0.05 compared to the normal skin tissue, and ** indicated *p* ＜ 0.01 compared to the normal skin tissue.

We analyzed changes in the expression of snRNAs in NST and KST. The sequence of snRNAs showed different expressions in KST and NST. Bioinformatics analysis revealed that compared with NST, the expression of seven snRNAs was upregulated significantly, and the expression of one snRNA was downregulated significantly, in KST. The number of variant snRNAs identified is less than the other ncRNAs, probably because of the smaller number of its species, as well as its more stable nature and thus less variable properties (Figures 3A–D). PCA showed that the snRNA expression in KST and NST had significantly different characteristics in both PC1 (26.1%) and PC2 (23.65%) (Figure 3E). The low number of snRNA variants in the keloid and the lack of significant clustering in PCA predict that it may not play a major role in the regulation of this disease. RT-qPCR revealed that expressions of ENSG00000200972 and ENSG00000201317 were upregulated in KST compared with that in NST (Figure 3F).

### Aberrant Expression of miRs Within the Keloid Skin Tissue

**FIGURE 4:**
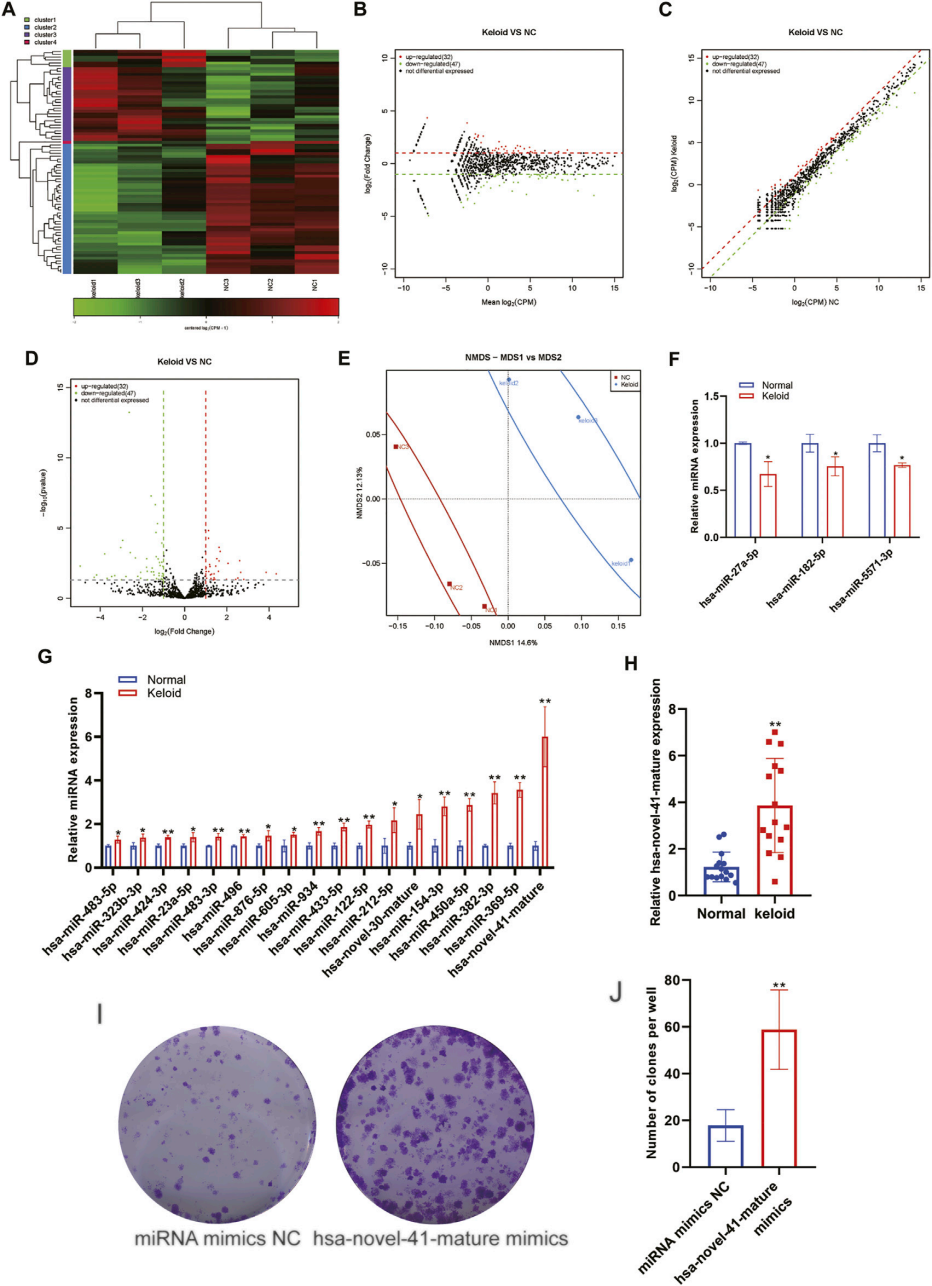
Aberrantly expressed miRNA within the keloid tissue. **(A)** Heatmap showed the expression of miRNA within the keloid tissue and normal tissue. **(B)** MA map showed the expression of miRNA within the keloid tissue and normal tissue. **(C)** Scatter map showed the expression of miRNA within the keloid tissue and normal tissue. **(D)** Volcano map showed the expression of miRNA within the keloid tissue and normal tissue. **(E)** PCA analysis of the expression of miRNA within the keloid tissue and normal tissue. **(F)** qPCR detection of the expression of miRNA downregulated in the keloid tissue and normal tissue. **(G)** qPCR detection of the expression of miRNA upregulated in the keloid tissue and normal tissue. * indicated *p* ＜ 0.05 compared to the normal skin tissue, and ** indicated *p* ＜ 0.01 compared to the normal skin tissue.

An miR is a type of endogenous small RNA of length 20–24 nucleotides. miRs have various important regulatory roles in cells. A complex network regulates the expression of multiple genes through one miRNA and also finely regulates the expression of a gene through a combination of several miRs. Some scholars have reported the miR expression in KST (Lv et al., 2020). We identified a total of 1323 distinct miRNAs and 120 novels miRNA. The detected miRNA was transcribed from intron (62%) and exon (38%). The most detected miRNA family is mir-548 which is highly expressed in the muscle tissue. The first base in both groups shows a similar base bias. There is no significant bias in strand selection (Supplementary Figure S1). We analyzed changes in the expression of miRs in NST and KST. The sequence of small ncRNAs showed different expressions of miRs in KST and NST. Bioinformatics analysis revealed that compared with NST, the expression of 32 miRs was upregulated significantly, and the expression of 47 miRs was downregulated significantly, in KST, with the relatively similar fold changes but higher credibility in downregulation (Figures 4A–D). PCA demonstrated that the mIR expression in KST and NST had significantly different characteristics (Figure 4E). RT-qPCR showed that the expression of three miRs was downregulated (Figure 3F), and the expression of 18 miRs was upregulated, in KST compared with that in NST (Figure 3G). The GO analysis reveals that the cytoplasm is highly unregulated between two groups; several related gene pathways might get involved such as the KEGG, which concludes the cGMP-PKG, MAPK signal pathway, and beta-Alanine metabolism are involved.

### Aberrant Expression of Repeat-Associated Small Interfering (rasi) RNAs Within Keloid Skin Tissue

The mechanism of RNA silencing is different in small interfering (si) RNAs and miRs (1). rasiRNAs are not dependent upon DICER1 or DICER2. The former is necessary for the miR formation, and the latter is needed for siRNA formation. Unlike miRs and siRNAs, siRNAs lack 2′,3′ hydroxyl terminals. Unlike miRs and siRNAs, which need Ago to perform functions, rasiRNAs need PIWI instead of Ago protein. However, no study has reported the rasiRNA expression in KST.

**FIGURE 5:**
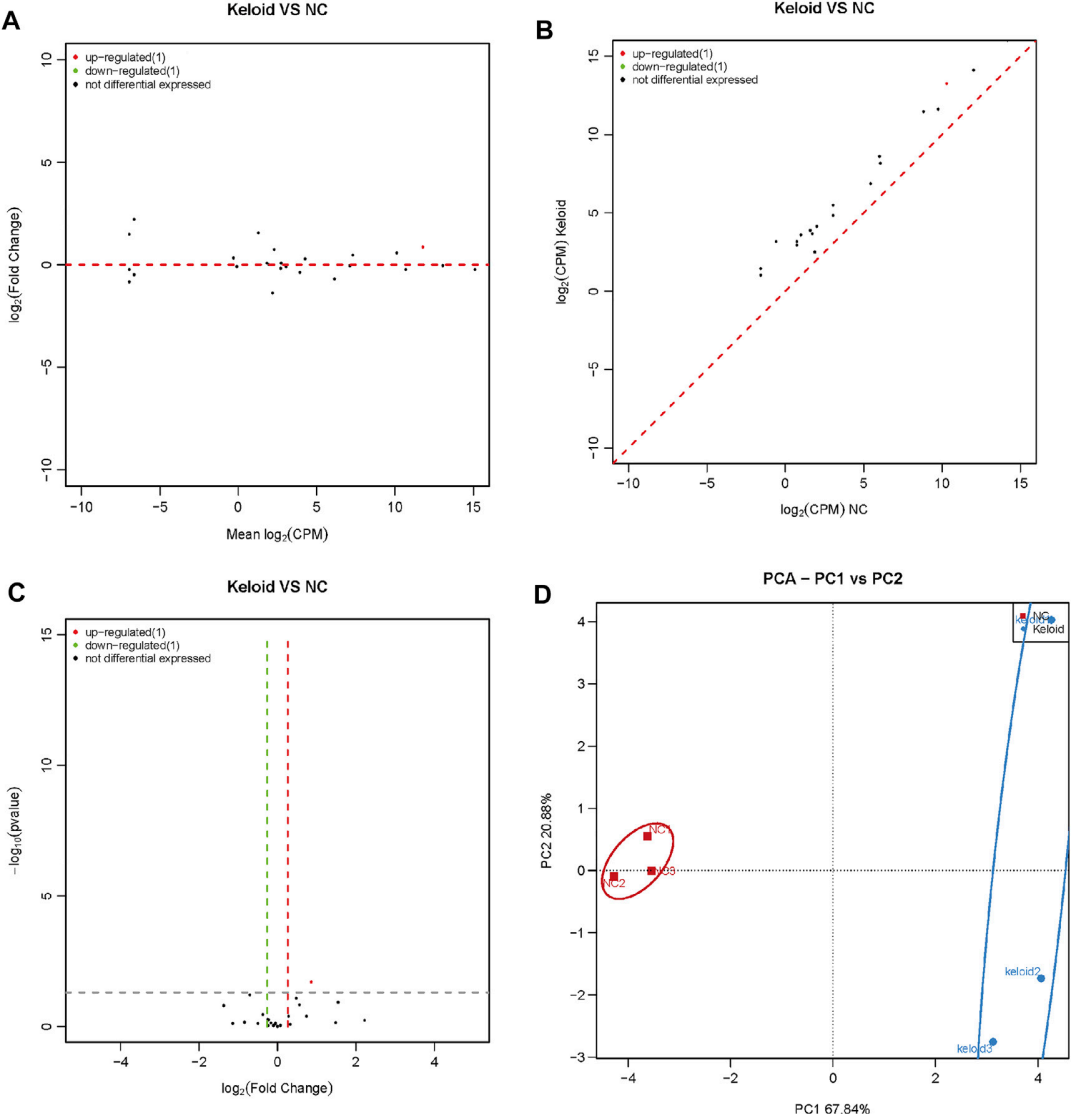
Aberrantly expressed rasiRNA within the keloid tissue. **(A)** MA map showed the expression of rasiRNA within the keloid tissue and normal tissue. **(B)** Scatter map showed the expression of rasiRNA within the keloid tissue and normal tissue. **(C)** Volcano map showed the expression of rasiRNA within the keloid tissue and normal tissue **(D)** PCA analysis of the expression of rasiRNA within the keloid tissue and normal tissue.

We analyzed the changes in the expression of rasiRNAs in NSTs and KSTs. The sequence of small ncRNAs showed different expressions of rasiRNAs in KST and NST. Bioinformatics analysis revealed that compared with NST, the expression of one rasiRNA was upregulated significantly, and the expression of one rasiRNA was downregulated significantly, in KST (Figures 5A–C). PCA showed that miR expressions in KST and NST had significantly different characteristics (Figure 5D).

## Discussion

Currently, the etiology and formation of keloids remain elusive. Many noncoding RNAs have been found to be involved in regulation in many diseases in recent years, and many studies have elaborated on noncoding RNA research in keloids, but their research on noncoding RNA is still limited in miRNA, lncRNA, and circRNA. Here, we investigated the “landscape” of the sncRNA expression in KST. In detail, we newly revealed the expression profiles of piRNA, snoRNA, snRNA, and repeat RNA in keloids and compared their expression characteristics with miRNA. We found that these RNAs have different expression characteristics. Compared with miRNA, piRNA exhibits higher fold changes and wider distribution variation, while snoRNA and snRNA exhibit less variation in the relatively insignificant expression difference between the two groups; we found only a few repeaters in the two groups. Functional prediction showed that these RNAs involved processes such as leukocyte transendothelial migration and lipolysis, and significant activation of PKG and MAPK signaling pathways. Some specific differentially expressed RNAs were screened and verified by PCR.

sncRNAs are a subset of epigenetic modifiers in various fibrosis-related diseases (Czech et al., 2018; Lu and Rothenberg, 2018). We employed sequencing of small RNAs to identify alterations in the expression of sncRNAs in NST and KST. We chose clinical tissue specimens because of an absence of an optimal model to simulate keloid formation completely (Rippa et al., 2019). Unlike tumor resection, which requires an expanded resection margin to avoid recurrence, the margin should be within the keloid to avoid damaging surrounding the normal skin and leading to keloid recurrence (Lee and Jang, 2018). Thus, obtaining NST from the same keloid patient is challenging. We chose the normal abdominal skin from abdominoplasty because skin tension, developmental source, and thickness closely resemble those of the chest skin (Kanitakis, 2002).

Fibroblasts have been recognized as the major effector cells in keloid formation, but many other cells that account for less proportion of keloid cells have been found to be important in keloid formation in recent years, such as M2 type macrophages, which promote fibroblast-to-myofibroblast transition (Shook et al., 2018), and keratinocytes, which participate in epithelial-to-mesenchymal transition (EMT) (Ma et al., 2015). From clinical experience, some keloids were induced from an injury only deep to the epidermis, which drew our attention to keratinocytes. We preliminarily compared the expression of some small noncoding RNAs between fibroblasts and keratinocytes (Supplementary Figure S1). The results also showed a promising role of keratinocytes in keloid formation.

The specific regulatory function of several mIRs has been determined: miR-21 targets SMAD to regulate cell proliferation (Wu et al., 2019), miR-29 regulates the expression of collagen-1α (Zhang et al., 2016), and miR-141 represses the GAB1 expression to attenuate cell migration (Feng et al., 2017). The miR expression in keloid fibroblasts, as well as fetal and adult normal skin, has been measured: similar expression in keloids and fetal normal skin has been documented (Li et al., 2013). Hence, miRs may be involved in fibroblast dedifferentiation in keloids. Several studies have found some specific miRs to be implicated in wound healing and keloid formation, such as miR-21 (Hu et al., 2018), miR-141 (Liang et al., 2020), and miR-200 (Zhou et al., 2019). Here, we annotated and validated multiple novel differential miRNAs in keloids, such as mir-202 and mir-41, two significantly differentially expressed miRNA subsets. miR-548 and miR-154 are the most counted miRNA families, and miR-21 and miR-199 have the most total reads. The regulatory role of miR-21 in wound healing has been widely studied, but the miR-199 in scar formation is still vacant. A study revealed that hypoxia-inducible factor 1 alpha (HIF1A), which is a wound abnormal healing–related factor, appeared to be the potential target of miR-199.

Unlike miRs, piRNAs are a very diverse category of ncRNAs. piRNAs identified by sequencing have little in common and classification is challenging (Huang et al., 2017), so they could have a different regulatory mechanism compared with that of miRs. piRNAs have been demonstrated to regulate transposons in germline cells by forming a RISC with PIWI proteins. G.J. Hannon and colleagues found that piRNAs can regulate gene expressions in somatic cells (Czech et al., 2018). The specific functions of piRNAs are not known, but some research progress has been made. The PIWI–piRNA system is part of tissue regeneration in salamanders (Kim et al., 2018). PIWI protein could be involved in cell differentiation; it shows higher expression in skin appendages (e.g., sebaceous glands) compared with that in compartments in which stem cells are located (Pammer et al., 2020). Here, we annotated multiple novel differential piRNAs in keloids, such as piR-38252 and hsa_piR_016659, two significantly differentially expressed piRNA subsets. But limited by the current study of piRNAs, the roles of these differentially expressed piRNAs in keloid formation need future research. Compared to the variation of miRNAs, piRNAs in keloids show an average of higher expression level, the individual fold change of piRNAs was significantly larger, and the number of low expressions was more than that of high expression.

Emerging evidence has demonstrated snoRNAs to be involved in cellular development and homeostasis (Bouchard-Bourelle et al., 2020). Studies have shown that the expression of non-canonical snoRNA is regulated in osteoarthritis, which is characterized by ECM disruption (Steinbusch et al., 2017). Excess deposition of the ECM has been observed in keloids, so snoRNAs may have roles in keloids. We annotated and validated multiple novel differential snoRNAs in keloids, such as SNORA20 and SNORA5C. Compared to piRNA and miRNA, the average expression of snoRNAs is higher and concentrated at around 1000 CPM, but there was no significant difference in the expression profile of snoRNA between the keloids and the control groups. The snRNA presents a similar expression profile to snoRNA.

## Data Availability

The datasets presented in this study can be found in online repositories. The name of the repository, accession number, and link can be found at: Â ArrayExpress; E-MTAB-11336; https://www.ebi.ac.uk/arrayexpress/experiments/E-MTAB-11336.
