# Supplementary material for: Aberrantly Expressed Small Noncoding RNAome in Keloid Skin Tissue
Source: Front Genet. 2022 Apr 13;13:803083. doi: 10.3389/fgene.2022.803083 (PMC9045488; doi:10.3389/fgene.2022.803083)
Supplement: Supplementary file 3 [file DataSheet5.PDF]

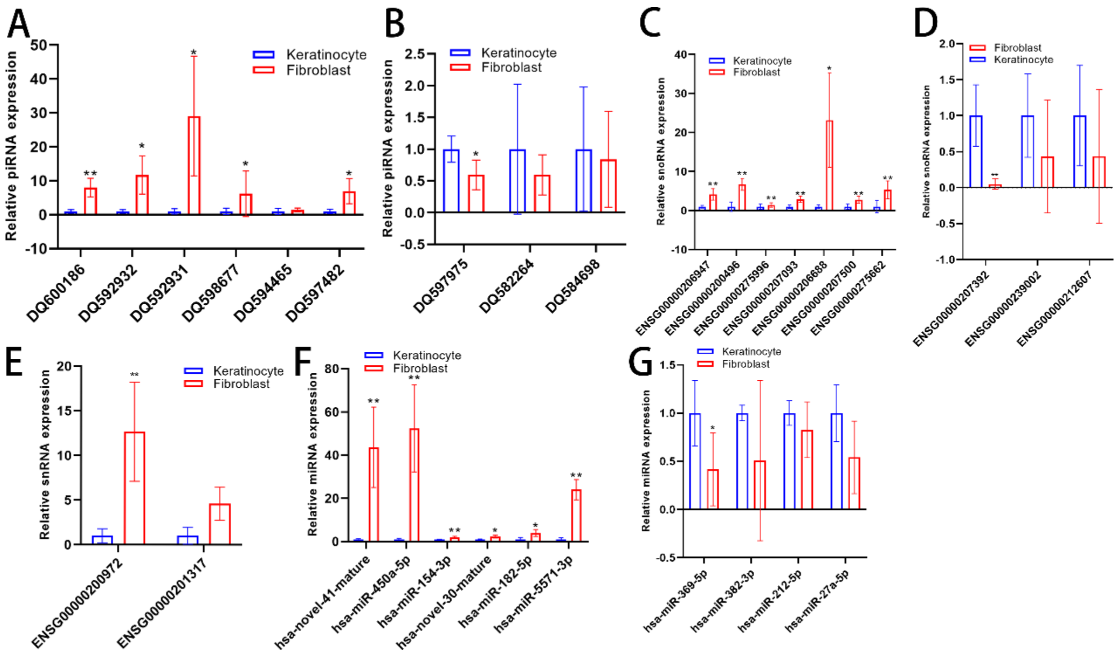

Supplement Figure 1 qPCR detection of the expression of sncRNA in fibroblast and keratinocyte from keloid tissue. (A) The piRNAs expressed higher in fibroblasts. (B) The piRNAs expressed higher in keratinocytes. (C) The snoRNAs expressed higher in fibroblasts. (D) The snoRNAs expressed higher in keratinocytes. (E) The snRNAs expressed higher in fibroblasts. (F) The miRNAs expressed higher in fibroblasts. (G) The miRNAs expressed higher in keratinocytes. Data showed with mean ±S.D. "*" indicated $P < 0.05$, "**" indicated $P < 0.01$.
